# Systematic development of an evidence-based website on preconception care

**DOI:** 10.1080/03009734.2016.1216481

**Published:** 2016-08-30

**Authors:** Ilse Delbaere, Pascale Mokangi, Kristien Roelens, An De Sutter, Xavier Gellynck, Dimitri Beeckman, Liselot van de Walle, Pieter Vandenbulcke, Hans De Steur

**Affiliations:** a Department of Midwifery, Health Education, Vives University College, Kortrijk, Belgium;; b Department of Obstetrics and Gynecology, Ghent University Hospital, Ghent, Belgium;; c Department of Family Medicine and Primary Health Care, Ghent University, Ghent, Belgium;; d Department of Agricultural Economics, Faculty of Biosciences Engineering, Ghent University, Ghent, Belgium;; e University Centre for Nursing and Midwifery, Department of Public Health, Ghent University, Ghent, Belgium;; f Public Health Agency ‘Zorg & Gezondheid’, Flemish Government, Brussels, Belgium

**Keywords:** Evidence-based, folic acid, guideline, information, preconception care, prevention

## Abstract

**Introduction:**

In February 2015, the Flemish Minister of Welfare, Public Health and Family launched a website on preconception care: ‘gezondzwangerworden.be’. The website was developed in response to the lack of comprehensive communication on preconception care and the inadequate intake of folic acid among Flemish women. Despite the international recommendation to take 400 μg folic acid on a daily basis one month before conception until 12 weeks of pregnancy, studies show a lack of compliance in women wanting to become pregnant.

**Procedure:**

A compilation of evidence was made through reviewing well-established guidelines on preconception and prenatal care. The quality of guidelines was assessed by means of AGREE II. The topics included in the website were selected by an internal committee of 5 experts and an external committee of 16 experts. Content validation was carried out by 40 experts in preconception care or related topics.

**Results:**

The above-described procedure resulted in an evidence-based website with a selection of relevant, validated information for both women and men who plan a pregnancy and professionals who are consulted by these people.

**Evaluation and recommendation:**

The website is currently attracting a constant number of 100 to 200 visitors a day. The information on folic acid is among the most requested, which is an important finding with regard to the policy objectives on preconception care. More research is needed in order to evaluate the use and effect of the website more thoroughly.

## Introduction

In February 2015, the website on preconception care, ‘gezondzwangerworden.be’, was launched by the Flemish Minister of Welfare, Public Health and Family. The website is edited in Dutch and holds evidence-based information for both women and men planning a pregnancy and health care providers.

Since the MRC Vitamin Study Research Group in 1991 found that a daily dose of 400 μg folic acid reduced the risk of neural tube defects drastically, periconceptional supplementation of folic acid has been recommended internationally to women planning a pregnancy (1). However, the time-frame in which women are advised to take folic acid differs between countries (2). The ‘Superior Health Council’ in Belgium recommends intake one month before pregnancy until pregnancy week 12; nonetheless, a study in 2010 on the practice of periconception folic acid intake in Flanders (the northern, Dutch-speaking part of Belgium) shows that only 36% of women actually pursue this recommendation (3).

Because of the proven effect of folic acid in the prevention of neural tube defects and recommendations of public and health authorities, on the one hand, and the lack of compliance in women planning a pregnancy, on the other hand, a resolution proposal on folic acid intake before conception and during pregnancy was granted by the Flemish Parliament on 16 June 2010 (4).

Subsequently, several stakeholders were involved in a number of meetings to discuss folic acid intake in the periconceptional phase. The public health agency ‘Zorg & Gezondheid’ and the Royal Academy for Medicine of Belgium organized these meetings on the initiative of the Flemish Minister of Welfare, Public Health and Family. After the final meeting on 28 April 2011, a public procurement was released on folic acid intake and preconceptional advice. One of the goals of this procurement was the development of an evidence-based website on preconception care, with a particular focus on folic acid intake. A multidisciplinary team from Ghent University worked on this procurement from April 2013 to July 2014. The website was developed in a systematic and scientific manner, and numerous experts were involved in the content validation.

## Procedure

### Search for and selection of guidelines

The content of the website is based on available guidelines on preconception and prenatal care. We searched in PubMed, Cochrane, Web of Science, Google, and Google Scholar using the following keywords: guideline(s), preconception, preconception care, preconception health, prepregnancy, before pregnancy, and before conception. We also visited international and national websites of professional organizations providing guidelines. Few guidelines on preconception care in healthy women were available. Some of the prenatal guidelines included information on the periconceptional phase, and some issues of the prenatal guidelines were relevant for preconception care as well. The guidelines perused were national guidelines from Domus Medica (Association of General Practitioners) (5) and KCE (Federal Centre of Knowledge for Public Health) (6), as well as international guidelines from CDC (www.cdc.gov/ncbddd/folicacid/recommendations.html), NHG (7), AJOG (8), ICSI (9), and NICE (10). The quality of the guidelines was assessed by three authors by means of AGREE II (the Appraisal of Guidelines for Research and Evaluation instrument is recognized as the gold standard for practice guidelines evaluation) (11).

### Selection of topics

All relevant topics of the selected guidelines were inventoried. The list was reviewed by a multidisciplinary committee of five internal experts (a gynaecologist, a general practitioner, two midwives, and a researcher with expertise on folic acid), and a first selection was made by this internal expert committee (IEC) (*n* = 5). Within the IEC, there was a consensus to include information on the items summed up in Table 1.

**Table 1.** Categories selected by the internal experts.

Categories	Subcategories
Nutrition	Folic acid and other micronutrients
Fertility and menstrual cycle	
Lifestyle	Legal and illegal drugs, body mass index, sports and leisure, hyperthermia, travelling, dental hygiene
Infections	Toxoplasmosis, listeriosis, STIs, hepatitis B, hepatitis C, HIV, CMV
Vaccinations	Rubella, varicella, pertussis
Anamnesis	Diabetes mellitus, epilepsy, asthma, thyroid dysfunction, hypertension, thrombo-embolic conditions, mental disorders, medication
Familial anamnesis	Genetic disorders, congenital malformation
Social factors	Domestic violence and information for the partner (drugs, hyperthermia, medication, harmful effects and radiation, STIs)
Other	Harmful effects of paint, hair dye, and radiation

In the selection of topics, it was our goal to focus on preconception care rather than prenatal or antenatal care. Topics considered less relevant for the website on preconception care were: information on breastfeeding, information on salmonella and parvovirus, soil and water pollution, personal hygiene, information on natal and postnatal care, pregnancy-related conditions, breast and abdominal examination, bacterial vaginosis, group B streptococcus, and asymptomatic bacteriuria.

Subsequently, the list of topics was sent to an external multidisciplinary committee of experts (EEC) (*n* = 16) together with a questionnaire where the experts could indicate if they agreed with the inclusion of information on the topic within the website and where they could mark the level of importance of every topic. Furthermore, the experts provided relevant information. Sixteen external experts were involved in the selection of topics: six general practitioners with expertise in preconception care, four gynaecologists (two academic and two non-academic), three midwives with expertise in preconception care, one pharmacologist, one nutritional expert, and one staff member of the most important Flemish public service for the well-being of children.

Table 2 depicts the advice of the EEC on the degree of importance for each topic to be included on the website intended for the public and professionals.

**Table 2.** Advice of external experts for topics to include in each part of the website. Number of experts (*n* = 16) indicating level of importance.

	Part of the website for the public			Part of the website for professionals		
	Very important	Important	Less important	Very important	Important	Less important
Folic acid	13	3	0	9	6	1
Smoking	10	6	0	8	8	0
Nutrition	6	9	1	3	10	1
Toxoplasmosis	5	8	2	5	8	2
Listeriosis	3	6	6	3	7	5
Alcohol	4	11	1	5	10	1
Drugs	5	10	1	5	9	1
Physical exercise	2	11	3	1	9	3
Low BMI	0	12	1	1	12	1
High BMI	1	14	0	1	11	2
Travelling	1	5	7	1	7	6
Hyperthermia	0	10	3	0	9	5
Oral hygiene	2	9	1	2	8	3
Occupational issues	3	8	1	1	10	3
Radiation	3	7	1	2	9	4
Paint	1	5	4	0	6	7
Socio-economic factors	5	7	1	5	9	1
Fertility	3	11	2	3	6	4
General anamnesis	2	9	1	4	10	1

As for healthy nutrition, experts indicated that basic information is known by professionals. Information on nutrition advice after bariatric surgery, malabsorption, and vitamin D is needed more for this group. According to the experts, it was necessary to provide information on women at risk of having a child with neural tube defects.

The few experts who did not consider it important to inform the public about the risk of toxoplasmosis reported that—in their opinion—the women were already sufficiently aware of the risk; however, most experts considered it necessary to include information on toxoplasmosis prevention. Information on smoking was considered very important because of the potential health benefit in smoking cessation. Experts advised to refer to smoking cessation guidance on the website. Information on physical exercise was considered important in the prevention of obesity with its adverse effect on fertility and pregnancy outcome. Experts considered that information on the optimal age for childbearing was not always relevant for this website. They argued that when people seek information on preconception care, they cannot change their age. Nonetheless, most experts agreed to include information on maternal age.

The arguments of the external experts were further discussed within the internal expert committee (see below).

### Content development

Based on the final list of topics, evidence was selected from guidelines and reviews, and a first draft text was written. Arguments of the external committee and contradictions in the guidelines were discussed with the internal expert committee (IEC).

### Content validation

In total, another 40 experts were involved in the revision of the information on the website (a detailed list of experts can be found on the bottom of the page ‘gezondzwangerworden.be/over-de-website’). Because of the importance of this process, experts were carefully chosen after consultation with the commissioning party (Zorg & Gezondheid) and the IEC. Thus, a multidisciplinary group was chosen to revise the part of the contents corresponding to their field of expertise. As such, gynaecologists, general practitioners, and midwives with an expertise in preconception care were involved; as were specialists in smoking cessation, obesity, and genetics; nutritional specialists; pharmacists; members of public services on occupational health, sexually transmitted infections, and hygiene; and delegates of health insurance companies, federations of birth defects, and members of the Dutch health council.

All experts revised their part of the draft text thoroughly and formulated suggestions and corrections. The content was modified according to the feedback from the experts. In case of uncertainties, the external experts were consulted. Again, we kept our focus on information that was relevant for couples who were not pregnant yet, as the majority of Flemish women are followed up thoroughly by a health care professional once they are pregnant.

In the draft version, we advised that women should stop alcohol intake when they were pregnant. Because of this remark, other experts were consulted and more literature was reviewed. This resulted in a stricter message, on alcohol use, within the website (avoid alcohol when you want to become pregnant).

### Feedback sessions with the commissioning party

Work progress was discussed with the commissioning party (Z&G) on a three-monthly basis. Choices were motivated and adjusted if necessary. In these meetings, it was decided that the website would have two parallel parts: a part for the lay public and a part for professionals; however, everyone should have access to both parts. The style of writing was adjusted to the target audience, and the content differed according to specific information needs.

Furthermore, the name of the website was decided in these meetings as well as the structure of the website, the choice of experts, and the choice of eye-catchers on the front page (folic acid, alcohol, and smoking).

### Pilot study

A pilot study was carried out by the ‘thinking aloud’ method (12) in a group of 30 first degree midwifery students (all female) on the one hand, and 6 people of reproductive age on the other hand (5 female, 1 male). One researcher led the group discussion, and another researcher noted the remarks. Without further information, the participants were asked to look at the front page of the website and talk about their first impression. They were asked what target audience they believed the website was intended for and what idea they had of the purpose of the website. Subsequently, they were asked to formulate a preconception-related question. After some explanation about the development of the website, the participants were asked further to explore the website and to search for the answer to their question on the website. They were asked if the website was complete in their opinion, or if it was too exhaustive, if all information was clear, if the website was user-friendly, and if they thought the website was appealing.

For the participants of the pilot study, the target group was obvious, the main messages and the purpose of the website were clear, information was found swiftly, and the website was easy accessible, appealing, and structured. There were some remarks about the colour use (some participants thought the pink was too female, other participants thought the colour would not discourage men visiting the website). Furthermore, suggestions for the lay-out were made. There were some remarks on grammar, participants found a few inactive links, and the need to explain difficult words in the website was expressed. Participants commented on the ambiguous message about alcohol use (to avoid alcohol when you are thinking of getting pregnant and stop drinking alcohol once you know you are pregnant) and pointed out that symptoms of STIs were not always systematically given. Based on the input from both groups, the website was further refined and improved.

**Figure 1.**
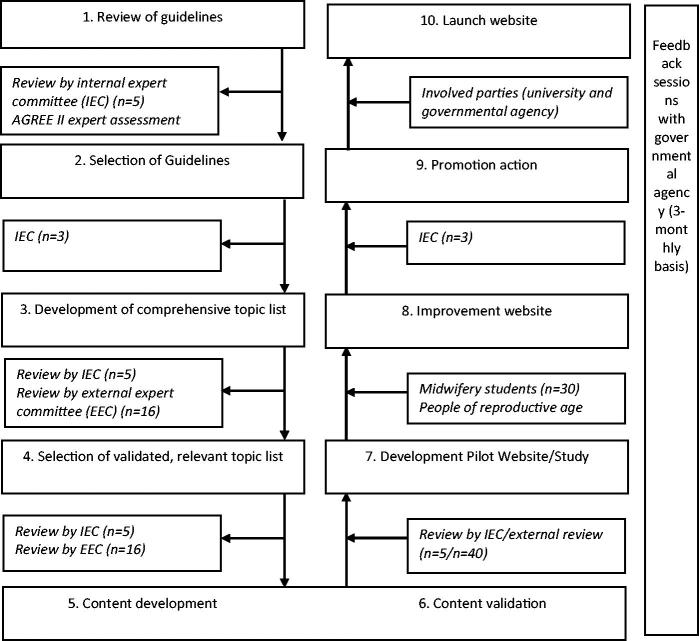
Flow of development process.

The development of the website is schematically depicted in Figure 1.

## Results

### The website

**Figure 2.**
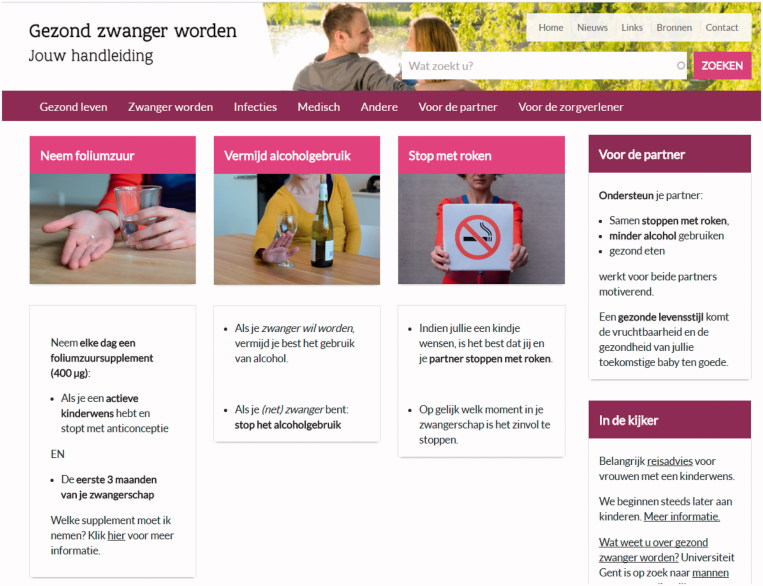
The website ‘gezondzwangerworden.be’.

The above-described process resulted in the website, ‘gezondzwangerworden.be’ (in English: ‘getting pregnant in good health’) (Figure 2). The front page holds information on folic acid, alcohol use avoidance, and smoking cessation. There is a direct link to information for the partner and a section where relevant news is updated regularly. Furthermore, there are seven main tab pages: ‘Healthy lifestyle’, ‘Becoming pregnant’, ‘Infections’, ‘Medical information’, ‘Other’, ‘For the partner’, and ‘For the professional’.

The tab page ‘Healthy lifestyle’ holds information on healthy nutrition, vitamins (folic acid and vitamin A), vegetarian and vegan nutrition, weight, hand and food hygiene, oral hygiene, alcohol, smoking, drugs, and leisure activities (physical activity, travelling, hyperthermia). The tab page ‘Becoming pregnant’ holds information on the menstrual cycle, lifestyle factors influencing fertility, and advice for people failing to conceive. On the tab page ‘Infections’, information is included on toxoplasmosis, listeria, rubella, varicella, pertussis, cytomegalovirus, parvovirus (this information was included later), STIs, hepatitis C, and tuberculosis. ‘Medical information’ holds information on personal anamnesis, medication, and vaccination. On the tab page ‘Other’, information is found on the domestic situation (socio-economic issues), occupational situation, cosmetics (hair dye and colourants, deodorants, and other), paint, and radiation. Furthermore, the tab page ‘For the partner’ holds information for both the male partner (lifestyle issues, such as alcohol, smoking, and drugs, hyperthermia, medication, harmful factors such as radiation, and information on STIs and hepatitis C) and information for the female partner (lifestyle issues and STIs). Finally, the tab page ‘For the professional’ has the same structure as the information for the public, but more information on medical issues is included: the information is more thorough and has other accents. For the professionals, the link to the source of information (guideline) is included within the text. The public can also look up the sources by means of a header ‘sources’ at the top of the website. Next to this header, there is a header ‘news’, where updates on the website are maintained and where relevant news on preconception care is found, a header where links to relevant websites are found, and a header ‘contact’ where visitors are encouraged to contact their professional for questions or to fill in a contact form if they want to provide feedback on the website.

The website was launched on 23 February 2015 by the Flemish Minister of Welfare, Public Health and Family, together with a press release. The news was covered in most written media and was mentioned in the television news at noon. The release of the website was announced to professional organizations of a large number of stakeholders (general practitioners, gynaecologists, midwives, health insurance companies).

## Evaluation

After one week, the website had attracted 8,850 users (there are approximately 30,000 births in Flanders per year to first-time mothers) of which almost 6,000 users visited the website on the day it was launched (in total we had 11,431 visitors during the first month). Most visitors (46%) were referred by news websites, 36% by direct entry, 10% by social media, and 8% by an organic search on search engines. We attracted users from all regions in Flanders.

The first week, we received 11 contact forms, of which 9 were from professionals. There was some feedback on the content, which was pursued when relevant. Some professionals asked for flyers to promote the website amongst their clients. Two care clients responded that the website was not easy to use on a smartphone or tablet, a problem which has been solved promptly by the webhost. After the first week and until now, only few contact forms were received.

Since this first week, which had much media coverage, and until today (a year after the launch) the website has been reaching a constant average of 100–200 visitors a day (more than 4,000 per month). Three out of four visitors were new, and 85% of the visitors were women. Table 3 shows the age distribution of visitors. People aged 25–34 years represented 62% of the visitors.

**Table 3.** Average age distribution of visitors to the website ‘gezondzwangerworden.be’.

Age category	Percentage of users
18–24 years	12.4%
25–34 years	62.4%
35–44 years	13.3%
45–54 years	7.6%
55–64 years	3.3%
65 years or older	1.1%

At the moment, 70% of visitors reach the website via an organic search on the internet (Table 4). People got to the website by searching on a variety of terms, such as ‘becoming pregnant’, ‘folic acid’, ‘healthy pregnancy’, ‘cycle’, and a combination of food products and ‘pregnancy’; 15% of visitors came via links from other websites. More than 77% of those visitors followed the link on the website of Kind & Gezin (kindengezin.be), which is the most important preventive organ for well-being of children in Flanders (the Flemish counterpart of ONE—l’Office de la Naissance et de l’Enfance). Amongst other things, people look at this website to find information on official childcare initiatives.

**Table 4.** Visitors to the website ‘gezondzwangerworden.be’.

Channel	Percentage of users
Organic search	70.5%
Referral (by other websites)	14.7%
Direct entry	14.2%
Social media	0.6%

The front page was, as expected, the most visited page of the website. After this, most users looked at the information on folic acid and then went to information on ‘healthy lifestyle’ and ‘becoming pregnant’ or ‘the menstrual cycle’.

## Updates

During this first year of existence, a number of updates have been carried out. First, contact information of a patient organization for people with an unfulfilled child wish has been added. Second, the information on vitamins and minerals has been updated according to the new nutritional recommendations of the Superior Health Council, and the information on ‘cosmetics’ has been extended due to a new report on anti-transpirants of this council. The advice of the same council to follow strict hygiene measurements in the prevention of CMV was added as well. Subsequently, a health care provider noticed the lack of information on parvovirus. After considering the evidence on this topic, information on the website was completed after review of three experts. Finally, information on the Zika virus was added with referral to an evidence-based and regularly updated website on tropical medicine (www.itg.be).

## Recommendations

Google analytics provide a service to evaluate the use of a website to some extent; however, the possibilities are limited. It is, for example, not clear how many and to what extent professionals refer to the website. Nonetheless, google analytics show that a number of visitors found our website by using a link provided on the website of professionals. On the contact form it is mentioned that specific questions can and should be discussed with the health care provider. Until now, the contact form was only used to provide feedback on the website (as is indicated on the website); however, it is not clear to what extent people address preconception care with their health care provider due to information they read on the website. Although the page with information on folic acid is one of the most visited pages of the website, it is currently not clear to what extent folic acid supplementation is (more) successful. It would be of interest to monitor folic acid intake for example in birth registers.

Finally, it would be valuable to publicize the website on a more regular basis, since an increased number of visitors is noticed each time the website is mentioned in the media (e.g. newspaper message).
